# Apoptosis, Proliferation, and Autophagy Are Involved in Local Anesthetic-Induced Cytotoxicity of Human Breast Cancer Cells

**DOI:** 10.3390/ijms232415455

**Published:** 2022-12-07

**Authors:** Jia-Lin Chen, Shu-Ting Liu, Shih-Ming Huang, Zhi-Fu Wu

**Affiliations:** 1 Institute of Medical Sciences, National Defense Medical Center, Taipei City 114, Taiwan; 2 Department of Anesthesiology, Tri-Service General Hospital, National Defense Medical Center, Taipei City 114, Taiwan; 3 Department of Biochemistry, National Defense Medical Center, Taipei City 114, Taiwan; 4 Department of Anesthesiology, Kaohsiung Medical University Hospital, Kaohsiung Medical University, Kaohsiung City 807, Taiwan; 5 Department of Anesthesiology, Faculty of Medicine, College of Medicine, Kaohsiung Medical University, Kaohsiung City 807, Taiwan; 6 Center for Regional Anesthesia and Pain Medicine, Wan Fang Hospital, Taipei Medical University, Taipei City 116, Taiwan

**Keywords:** triple-negative breast cancer, apoptosis, proliferation, autophagy, combination index

## Abstract

Breast cancer accounts for almost one quarter of all female cancers worldwide, and more than 90% of those who are diagnosed with breast cancer undergo mastectomy or breast conservation surgery. Local anesthetics effectively inhibit the invasion of cancer cells at concentrations that are used in surgical procedures. The limited treatment options for triple-negative breast cancer (TNBC) demonstrate unmet clinical needs. In this study, four local anesthetics, lidocaine, levobupivacaine, bupivacaine, and ropivacaine, were applied to two breast tumor cell types, TNBC MDA-MB-231 cells and triple-positive breast cancer BT-474 cells. In addition to the induction of apoptosis and the suppression of the cellular proliferation rate, the four local anesthetics decreased the levels of reactive oxygen species and increased the autophagy elongation indicator in both cell types. Our combination index analysis with doxorubicin showed that ropivacaine had a synergistic effect on the two cell types, and lidocaine had a synergistic effect only in MDA-MB-231 cells; the others had no synergistic effects on doxorubicin. Lidocaine contributed significantly to the formation of autophagolysosomes in a dose-dependent manner in MDA-MB-231 cells but not in BT-474 cells. Our study demonstrated that the four local anesthetics can reduce tumor growth and proliferation and promote apoptosis and autophagy.

## 1. Introduction

Breast cancer accounts for almost one quarter of all female cancers worldwide, and more than 90% of those who are diagnosed with breast cancer undergo mastectomy or breast conservation surgery. Breast cancer is clinically divided into three main subtypes: luminal estrogen receptor (ER)-positive and progesterone receptor (PR)-positive, which are further subdivided into luminal A and B; human epidermal growth factor receptor 2 (HER2)-positive; and 15% triple-negative breast cancer (TNBC), which accounts for 25% of all breast cancer-related deaths [1,2]. Responses to endocrine therapy and anti-HER-2 targeted therapy are poor, which results in limited treatment options for TNBC [1,3,4]. Recent findings have suggested that multiple genes are involved in the different types of TNBC and that their expression profiles are disease-specific, making it difficult to develop targeted therapies for all types of TNBC [1]. Given their high metastatic potential and lower responses to endocrine therapy and chemotherapy, various candidate small-molecule drugs, including single-target drugs and repurposed drugs, have emerged as promising TNBC therapies [1,3].

In clinical practice, breast cancer surgery can be carried out under general anesthesia with or without regional anesthesia. Previous studies have reported that local anesthesia is associated with longer recurrence-free periods for patients with breast cancers following surgical resection, but some of those studies have not presented any data to support the assertion that local anesthesia reduces cancer recurrence or improves cancer-related survival [5,6,7]. However, in a systematic review and network meta-analysis, Wong et al. reported that in breast cancer surgery, local anesthetic-based regional anesthesia modalities were preferable to control or local anesthetic infiltration from an analgesic perspective, demonstrating a clinically significant decrease in pain score and cumulative opioid consumption [8]. Local anesthetics have been proposed to cause the death of cancer cells via apoptotic and necrotic pathways [9,10]. Lidocaine is the most commonly used local anesthetic, and it effectively inhibits the invasiveness of cancer cells at the concentrations that are used in surgical procedures, including breast cancer surgery [11,12,13]. However, the underlying mechanisms of cytotoxicity in cancer cells have not been sufficiently elucidated.

Local anesthetics indirectly affect cancer biology, including the reduction of the demand for opioids [14]. Opioids are immunosuppressive, which may reduce patients’ resistance to tumor metastasis [15]. The co-administration of non-opioid and opioid adjuvants improves the latency of the onset and duration of analgesia and counteracts the disadvantageous effects of local anesthetics on cardiac dysfunctions. Furthermore, recent research on the use of local anesthetics in oncology has suggested that local anesthetics can be repurposed as chemosensitizers or synergistic therapies [16,17,18]. For example, lidocaine enhances the toxicity of some anticancer drugs, including mitomycin C, pirarubicin, and cisplatin [16,17]. Lidocaine also increases the sensitivity of breast cancer cells to tamoxifen by downregulating the expression of Golgi transport 1A, which enhances prognosis [18]. Based on “combination wisdom”, partner relationships between anticancer drugs and local anesthetics urgently need to be developed to increase the efficacy of anticancer drugs and decrease the side effects of anticancer drugs and local anesthetics for breast cancers, specifically TNBC.

The process of autophagy is the delivery of intracellular components and dysfunctional organelles to lysosomes for degradation and recycling. Autophagy maintains protein and organelle renewal, thereby ensuring homeostasis [19,20]. Autophagy consists of several sequential steps: initiation, membrane nucleation, elongation for autophagosome formation (maturation), and fusion with lysosomes to form autophagolysosomes for degradation and recycling. Recently, an increasing number of studies have shown the association between autophagy and cancer. However, autophagy plays a “double-edged sword” role in tumorigenesis [21,22,23]. Several lines of evidence have pointed to the role of autophagy in various stages of tumor metastasis in breast cancer [24,25]. However, a clear relationship between local anesthesia and the autophagy process within breast cancer is currently unestablished.

One recent study showed that most local anesthetics were toxic to cancer cells at high concentrations but that different anesthetics had different effects on the same tumor cell line, such as bupivacaine > lidocaine > ropivacaine [13]. In the present study, we sought to examine the cytotoxic effects of four local anesthetics, lidocaine, ropivacaine, levobupivacaine, and bupivacaine, on two breast tumor cell types, TNBC MDA-MB-231 cells and triple positive breast cancer BT-474 cells. We examined the potential cytotoxic effects of these four local anesthetics through analyses of cell cycle profiles, cellular proliferation, reactive oxygen species (ROS), autophagy, and combinatory therapy with doxorubicin. Our mechanistic findings could provide new insights into our understanding of the working mechanisms of popular local anesthetics in human breast cancer therapy, especially TNBC therapy.

## 2. Results

### 2.1. The Cytotoxic Effects of Lidocaine, Levobupivacaine, Bupivacaine, and Ropivacaine on Two Human Breast Cancer Cell Lines (MDA-MB-231 and BT-474)

**Figure 1:**
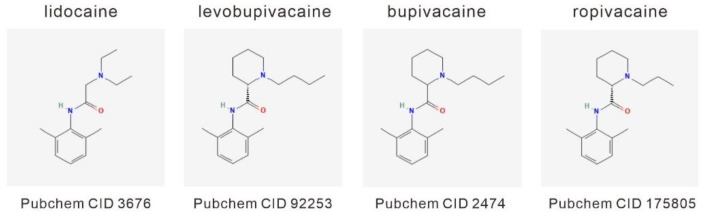
A diagram of the local anesthetics: lidocaine, levobupivacaine, bupivacaine, and ropivacaine. Compound ID (CID) is the identifier from a database of chemical molecules and their activities of biological assays in PubChem.

Local anesthetics are commonly used for dental and outpatient surgical procedures and the relief of localized pain. Two chemical structures, such as amide and ester types, of local anesthetics were developed; the former is metabolized by the liver and the latter is metabolized by plasma pseudocholinesterase, and their metabolites are excreted through urine [26]. Amide-type local anesthetics are more commonly used due to their better pharmacokinetic properties and lower incidence of adverse effects. Amide-type local anesthetics include lidocaine, levobupivacaine, bupivacaine, and ropivacaine (Figure 1).

**Figure 2:**
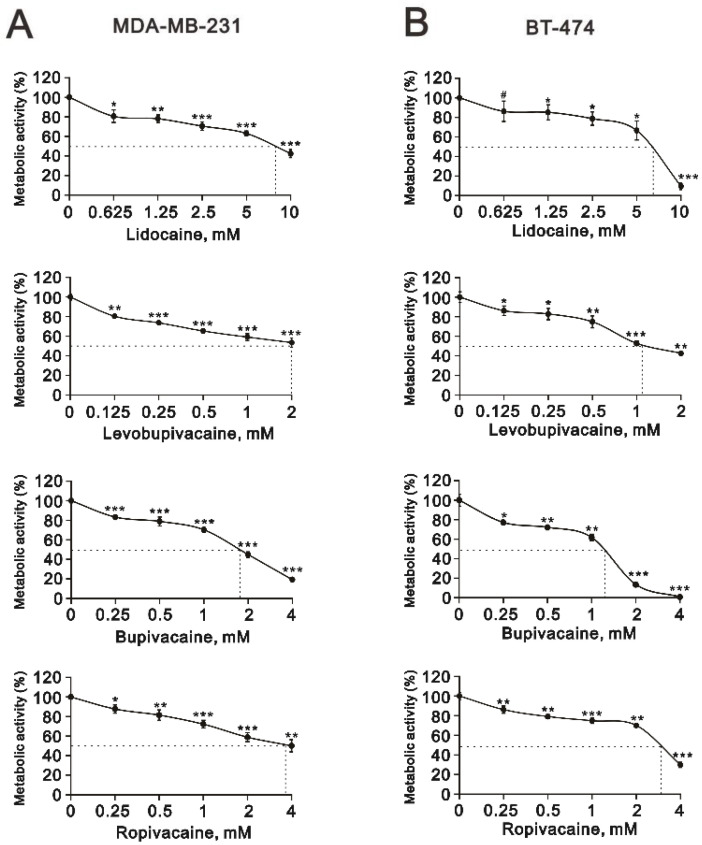
The effects of the local anesthetics on cell viability in MDA-MB-231 and BT474 cells. (**A**,**B**) MDA-MB-231 and BT-474 (8 × 10^4^) cells that were treated with the indicated concentrations of lidocaine (0.625, 1.25, 2.5, 5, and 10 mM), levobupivacaine (0.125, 0.25, 0.5, 1, and 2 mM), bupivacaine (0.25, 0.5, 1, 2, and 4 mM), and ropivacaine (0.25, 0.5, 1, 2, and 4 mM) for 24 h. Control cells were cultured under identical conditions. Metabolic activity was measured using an MTT colorimetric assay. Data are presented as a percentage of the control. Bars depict the mean ± SD. # *p* > 0.05; * *p* < 0.05; ** *p* < 0.01; *** *p* < 0.001.

The definition of a clear relationship between local anesthesia and the clinical outcomes of patients with breast cancer is an unmet medical need. Hence, we sought to examine the cytotoxic effects of four common local anesthetics, lidocaine, levobupivacaine, bupivacaine, and ropivacaine (Figure 1), on two breast tumor cell lines, MDA-MB-231 and BT-474. First, we applied an MTT metabolic activity assay to measure the effect of lidocaine, levobupivacaine, bupivacaine, and ropivacaine on cell viability in the MDA-MB-231 and BT-474 cell lines (Figure 2A for MDA-MB-231 cells; Figure 2B for BT-474 cells). The relative IC50 values were 8.5 mM for lidocaine, 2 mM for levobupivacaine, 1.8 mM for bupivacaine, and 3.8 mM for ropivacaine in MDA-MB231 cells (Figure 2A) and 6.5 mM for lidocaine, 1.1 mM for levobupivacaine, 1.3 mM for bupivacaine, and 3.2 mM for ropivacaine in BT-474 cells (Figure 2B). Overall, these local anesthetics were more cytotoxic to BT-474 cells than MDA-MB-231 cells. Local anesthetics are commonly used for dental surgical procedures; hence, we applied lidocaine, levobupivacaine, bupivacaine, and ropivacaine in one human normal gingival epithelial Smulow–Glickman (SG) cell to examine the relative cytotoxic effects. Compared with IC50 data: 6.5 mM for lidocaine, 1.1 mM for levobupivacaine, 1.3 mM for bupivacaine, and 3.2 mM for ropivacaine in BT-474 cells, the relative percentages of cell viability were 81 ± 3, 80 ± 1, 76 ± 2, and 78 ± 4 in SG cells treated with lidocaine, levobupivacaine, bupivacaine, and ropivacaine, respectively.

**Figure 3:**
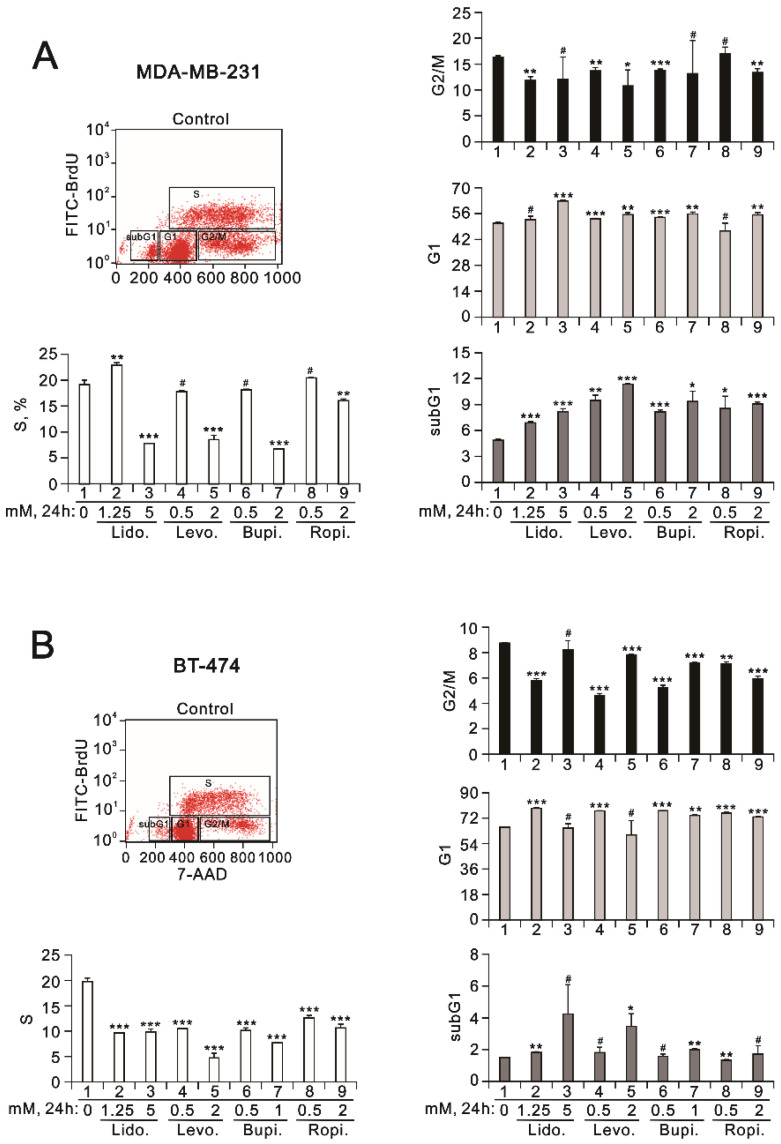
The effects of the local anesthetics on cell cycle profiles in MDA-MB-231 and BT-474 cells. (**A**) MDA-MB-231 and (**B**) BT-474 (3 × 10^5^) cells were treated for 24 h with the indicated concentrations of the local anesthetics. After treatment, the cells were stained with 7-Aminoactinomycin D (7-AAD), which is a dye that can bind to total DNA, coupled with immunofluorescent BrdU staining. Four cell subpopulations (i.e., subG1, G1, S, and G2/M phases) were distinguished based on bivariate BrdU incorporation and DNA content distribution (7-AAD staining): cells in the sub-G1 phase had a lower DNA content (hypodiploid); cells in the G1 and G2/M phases were DNA diploid and tetraploid, respectively; thus, the G2/M phase fluoresced twice as brightly and there was no BrdU incorporation; cells in the S phase had the highest and relatively constant BrdU levels. The 7-AAD staining intensities defined the cell cycle position. The cell cycle profiles were measured using flow cytometry analysis. The results are representative of two independent experiments. Bars depict the mean ± SD. # *p* > 0.05; * *p* < 0.05; ** *p* < 0.01; *** *p* < 0.001.

Cell viability is determined by cellular proliferation and cell death. We further examined the effect of the local anesthetics on cell viability by analyzing the cell cycle profiles and using a BrdU proliferation assay. In addition to the four local anesthetics increasing the population of subG phase, the population of the G1 phase was increased and the population of the G2/M phase was decreased in MDA-MB-231 cells (Figure 3A). For the S phase, 1.25 mM of lidocaine increased the population and 5 mM of lidocaine decreased the population, as did the other tested dosages of 2 mM of levobupivacaine, bupivacaine, and ropivacaine. In BT-474 cells, a decreased population of the S phase and an increased population of the G2/M phase were observed (Figure 3B). Increased populations of subG1 and G1 phases were also observed at various specific dosages of the local anesthetics.

**Figure 4:**
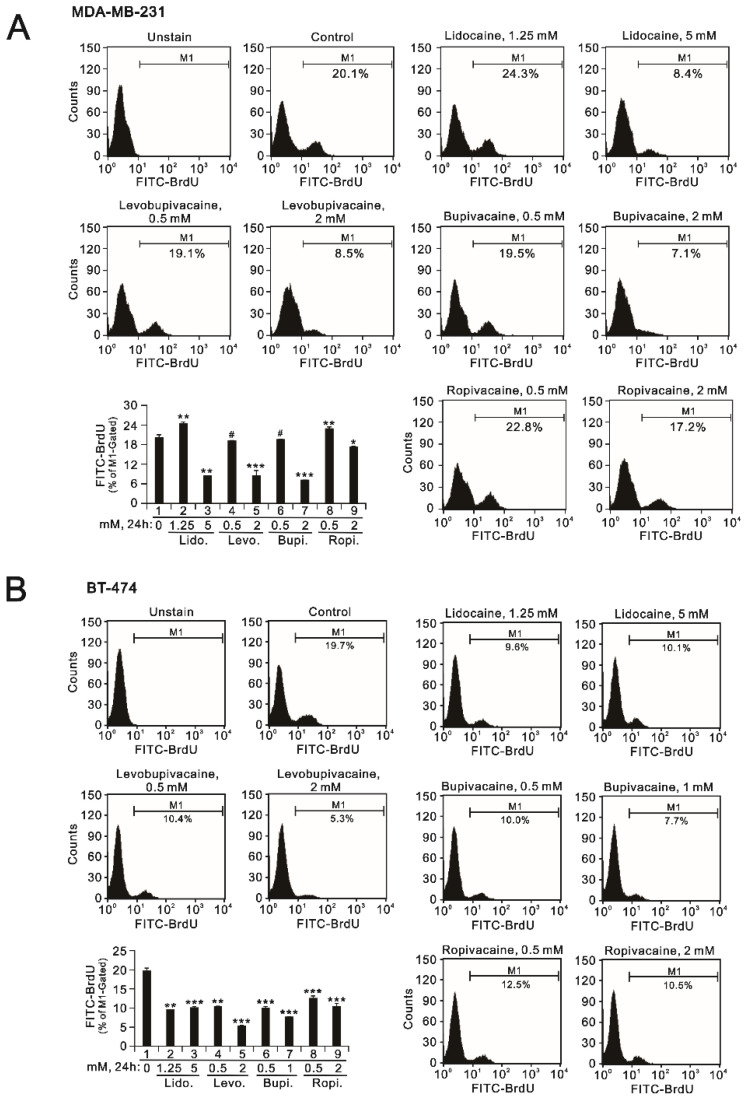
The effects of the local anesthetics on cell DNA synthetic activity in MDA-MB231 and BT-474 cells. (**A**) MDA-MB-231 and (**B**) BT-474 (3 × 10^5^) cells were treated for 24 h with the indicated concentrations of the local anesthetics. After treatment, the cells were stained with BrdU, incorporated into newly synthesized DNA, progressed through the S phase (DNA synthesis), and then measured using flow cytometry analysis relative to the BrdU incorporation level. The results are representative of two independent experiments. Bars depict the mean ± SD. # *p* > 0.05; * *p* < 0.05; ** *p* < 0.01; *** *p* <0.001.

Our BrdU proliferation data were consistent with the effects of the four local anesthetics on the populations of the S phase in MDB-MA-231 and BT-474 cells (Figure 4). In MDB-MA-231 cells, 1.25 mM of lidocaine and 0.5 mM of ropivacaine significantly elevated the cellular proliferation rate, and all tested anesthetics significantly reduced the cellular proliferation rate at the highest dosages (Figure 4A). In BT-474 cells, declining trends were observed under all testing conditions (Figure 4B).

**Figure 5:**
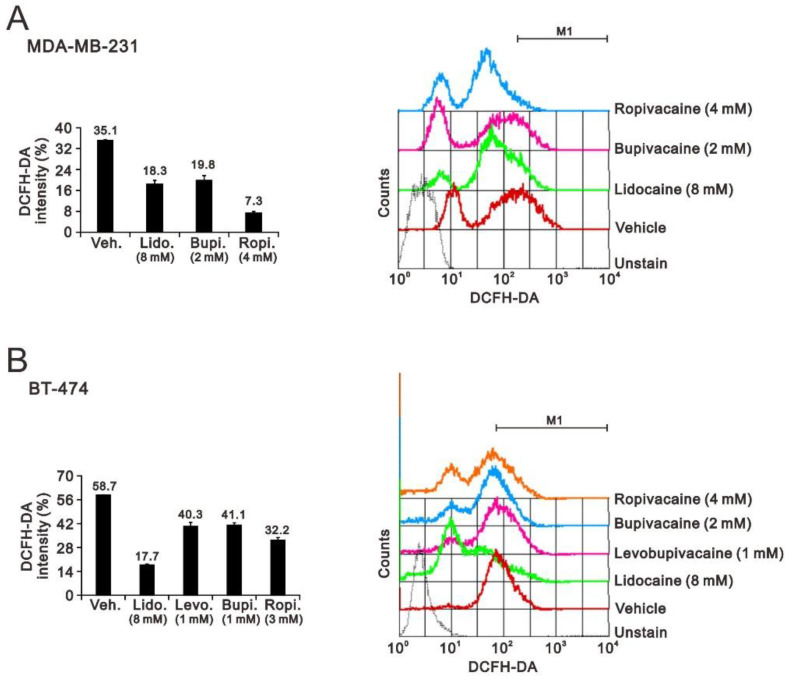
The effects of the local anesthetics on cell ROS status in MDA-MB-231 and BT474 cells. (**A**) MDA-MB-231 and (**B**) BT-474 (3 × 10^5^) cells were treated for 24 h with the indicated concentrations of the local anesthetics. The cell ROS status was determined using 20 µM of DCFH-DA and measured using flow cytometry. The cell volume gating strategy involved FSC-H and SSC-H. The median DCFH-DA fluorescence intensity of the vehicle was used as the starting point for M1 gating. The results are representative of two independent experiments. Bars depict the mean ± SD.

Oxidative stress results from an imbalance between the production of ROS and the antioxidant capacity [27]. Oxidative damage is not only a cause but also a consequence of various types of cell death. Elevated oxidative stress has been reported to be a potential anticancer treatment strategy in many studies [28,29,30,31,32]. Hence, we applied the IC50 concentrations of lidocaine, levobupivacaine, bupivacaine, and ropivacaine with DCFH-DA dye to measure the changes in ROS status in MDA-MB-231 and BT-474 cells. We failed to observe the ROS status of levobupivacaine in MDA-MB-231 cells because of the lethal dosage. The other anesthetics showed a decline in ROS status in MDA-MB-231 and BT-474 cells (Figure 5).

### 2.2. The Effects of Lidocaine, Levobupivacaine, Bupivacaine, and Ropivacaine on the Induction of Autophagy in MDA-MB-231 and BT-474 Cell Lines

**Figure 6:**
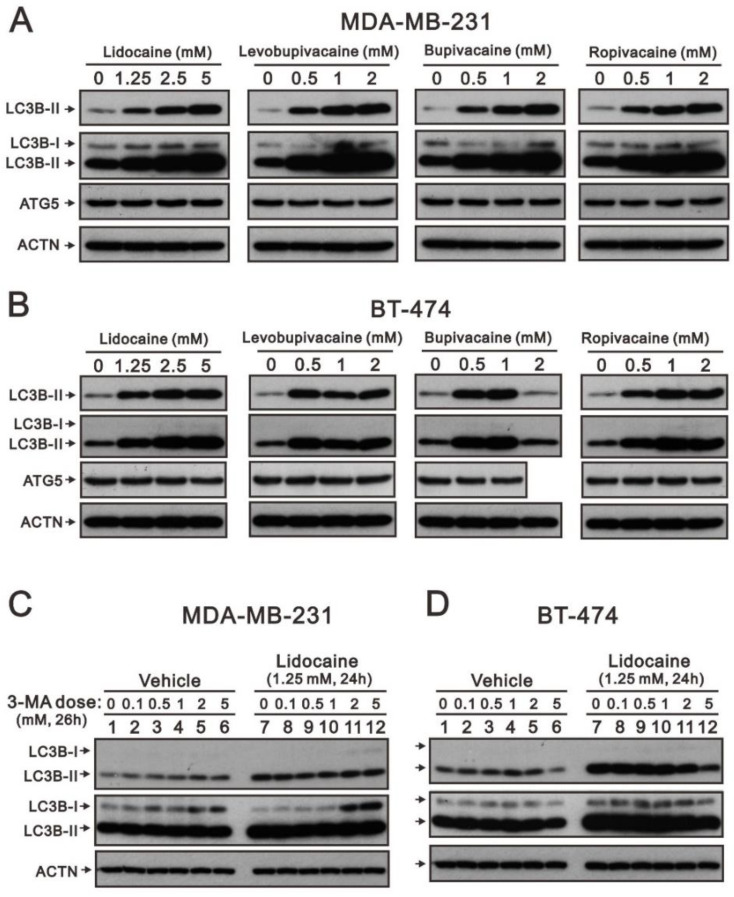
The effects of the local anesthetics on autophagosome-related protein expression in MDA-MB-231 and BT-474 cells. (**A**) MDA-MB-231 and (**B**) BT-474 (3 × 10^5^) cells were treated with the indicated concentrations of lidocaine (1.25, 2.5, and 5 mM), levobupivacaine (0.5, 1, and 2 mM), bupivacaine (0.5, 1, and 2 mM), and ropivacaine (0.5, 1, and 2 mM) for 24 h; (**C**) MDA-MB-231 and (**D**) BT-474 cells were pre-treated for 2 h with 0, 0.1, 0.5, 1, 2, and 5 mM of 3-MA and then combined with 1.25 mM of lidocaine for 24 h. The cell lysates were subjected to Western blotting analysis using antibodies against the indicated proteins. ACTN was used as the protein loading control.

At least three morphologically distinct cell death processes have been named: apoptosis, autophagic cell death, and necrosis [33,34]. Autophagy is the process of the delivery of intracellular components and dysfunctional organelles to lysosomes for degradation and recycling. The accumulation of microtubule-associated protein 1 light chain 3B II (LC3B II) and p62 increased after treatment with the autophagy blocker bafilomycin A1. Hence, we analyzed the ratio of LC3B II/I, which is a marker for the elongation step of autophagy, to determine whether autophagy was induced by lidocaine, levobupivacaine, bupivacaine, or ropivacaine in MDA-MB-231 and BT-474 cells. Our Western blotting data showed that lidocaine, levobupivacaine, bupivacaine, and ropivacaine increased the ratio of LC3B II/I in a dose-dependent manner in MDAMB-231 cells (Figure 6A). In BT-474 cells, only lidocaine and ropivacaine increased the ratio of LC3B II/I in a dose-dependent manner (Figure 6B). No effects of 2 mM of bupivacaine were observed on the ratio of LC3B II/I. Autophagy-related gene 5 (ATG5) as well as LC3B (ATG8) is a key component of autophagy to regulate the formation of the autophagosome. Our Western blotting analysis showed that no ATG5 protein level was disrupted by these local anesthetics (Figure 6A,B).

It is known that 3-methyladenine (3-MA) interferes with the formation of autophagosomes by inhibiting class III PI3K vPS34, which is the most common pharmacological agent used to suppress autophagy [35]. We chose to study lidocaine to verify its ability for the induction of autophagy via 3-MA in MDA-MB-231 and BT-474 cells. The effects of 3-MA alone were different in MDA-MB-231 and BT-474 cells. Overall, 3-MA decreased the ratio of LC3B II/I in MDA-MB-231 cells (Figure 6C) and decreased the amount of LC3B II in BT-474 cells (Figure 6D). With the autophagy induction by lidocaine, 3-MA consistently increased the ratio of LC3B II/I in MDAMB-231 cells (Figure 6C) and consistently decreased the amount of LC3B II in BT-474 cells (Figure 6D).

**Figure 7:**
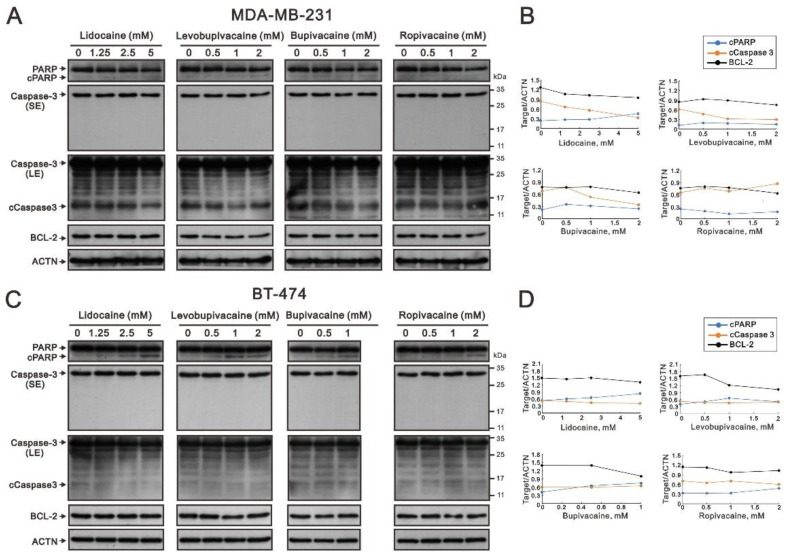
The effects of the local anesthetics on apoptosis-related protein expression in MDA-MB-231 and BT-474 cells. (**A**) MDA-MB-231 and (**C**) BT-474 (3 × 10^5^) cells were treated with the indicated concentrations of lidocaine (1.25, 2.5, and 5 mM), levobupivacaine (0.5, 1, and 2 mM), bupivacaine (0.5 and 1 mM), and ropivacaine (0.5, 1, and 2 mM) for 24 h. The cell lysates were subjected to Western blotting analysis using antibodies against the indicated proteins. ACTN was used as the protein loading control. SE: shorter exposure; LE: longer exposure. (**B**,**D**) The protein bands (**A**,**C**) were quantified through pixel density scanning and evaluated using ImageJ, version 1.44a (http://imagej.nih.gov/ij/) (accessed on 4 December 2022). The ratios of protein/ACTN, including cPARP, cCaspase 3, and BCL-2) were plotted. The molecular weight markers are labeled with kDa.

We observed that the subG1 populations were increased by lidocaine, levobupivacaine, bupivacaine, or ropivacaine in Figure 3. Hence, we further analyzed the cleaved status of caspase 3 and PARP proteins and BCL2, a protein that promotes cellular survival and inhibits the actions of pro-apoptic proteins, in MDA-MB-231 and BT-474 cells. We observed two pieces of apoptotic evidence that the amounts of cleaved PARP were increased or the amounts of BCL-2 were decreased by the tested local anesthetics (Figure 7A,C). In these two human breast cancer cell lines, a decrease in BCL-2 proteins was observed (Figure 7B,D, black lines) but it was hard to observe the cleaved form of caspase 3 (Figure 7B,D, cCaspase 3, orange lines). In addition, the cleaved form of PARP was indued by lidocaine, levobupivacaine, bupivacaine, and ropivacaine in BT-474 cells (Figure 7D, cPARP, blue lines) and was induced by lidocaine and bupivacaine in MDA-MB-231 cells (Figure 7B).

**Figure 8:**
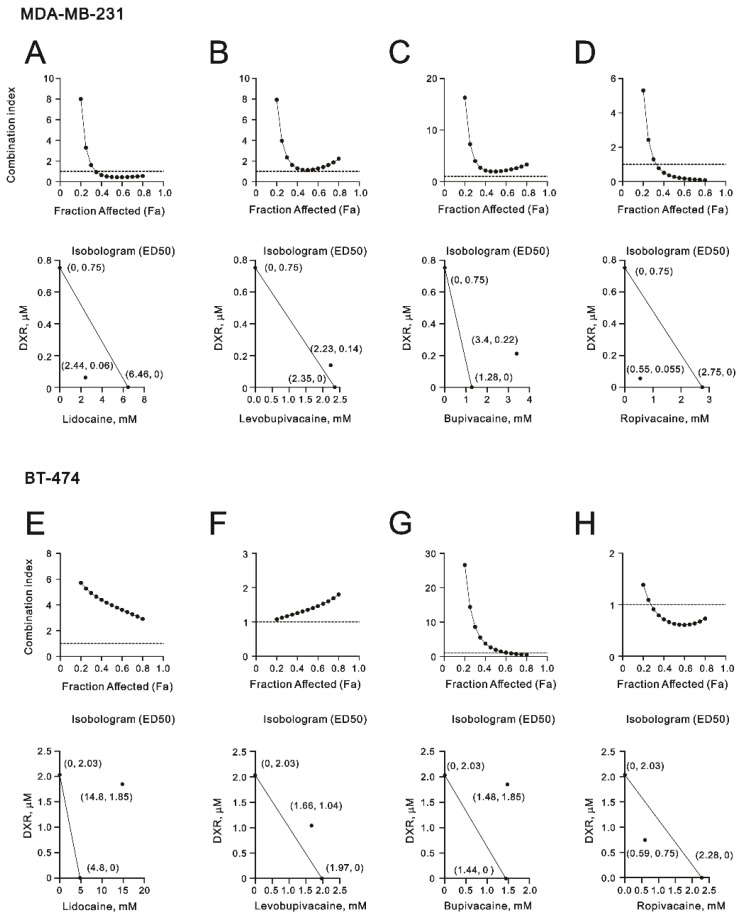
The combination indices of the local anesthetics with DXR in MDA-MB-231 and BT-474 cells. (**A**–**H**) The combination indices of DXR plus lidocaine, levobupivacaine, bupivacaine, and ropivacaine in (**A**–**D**) 1.5 × 10^4^ MDA-MB-231 cells and (**E**–**H**) 2.5 × 10^4^ BT-474 cells; (**A**–**D**) MDA-MB-231 cells were treated with various DXR doses (0, 0.01, 0.025, 0.05, 0.1, 0.25, 0.5, and 1 µM) combined with various lidocaine doses (0, 0.156, 0.3125, 0.625, 1.25, 2.5, 5, 10, 20, and 40 mM), levobupivacaine doses (0, 0.03125, 0.0625, 0.125, 0.25, 0.5, 1, 2, 4, and 8 mM), bupivacaine doses (0, 0.0156, 0.03125, 0.0625, 0.125, 0.25, 0.5, 1, 2, and 4 mM), and ropivacaine doses (0, 0.0625, 0.125, 0.25, 0.5, 1, 2, 4, 8, and 10 mM); (E,F, G, H) BT474 cells were treated with various DXR doses (0, 0.156, 0.3125, 0.625, 1.25, 2.5, 5, and 10 µM) combined with various lidocaine doses (0, 0.156, 0.3125, 0.625, 1.25, 2.5, 5, 10, 20, and 40 mM), levobupivacaine doses (0, 0.03125, 0.0625, 0.125, 0.25, 0.5, 1, 2, 4, and 8 mM), bupivacaine doses (0, 0.0156, 0.03125, 0.0625, 0.125, 0.25, 0.5, 1, 2, and 4 mM), and ropivacaine doses (0, 0.03125, 0.0625, 0.125, 0.25, 0.5, 1, 2, 4, and 8 mM) for 24 h. Metabolic activity was measured using the MTT method. The isobolograms (ED50) of lidocaine, levobupivacaine, bupivacaine, and ropivacaine were calculated using CalcuSyn software.

The roles of local anesthetics are not independent, and these agents are more likely to be used as chemo-sensitizers or synergistic therapies. Hence, we examined lidocaine, levobupivacaine, bupivacaine, and ropivacaine combined with the common chemotherapy drug doxorubicin using a combination index analysis of MDA-MB-231 and BT-474 cells (Figure 8A–H). Our data showed that for a combination index < 1, a synergistic effect was observed from the combination of ropivacaine and doxorubicin in MDA-MB-231 and BT-474 cells (Figure 8D,H) and from the combination of lidocaine and doxorubicin in MDA-MB-231 cells (Figure 8A). The concentration of doxorubicin with ropivacaine decreased from 0.75 µM to 0.55 µM in MDA-MB-231 cells and from 2.03 µM to 0.75 µM in BT-474 cells.

**Figure 9:**
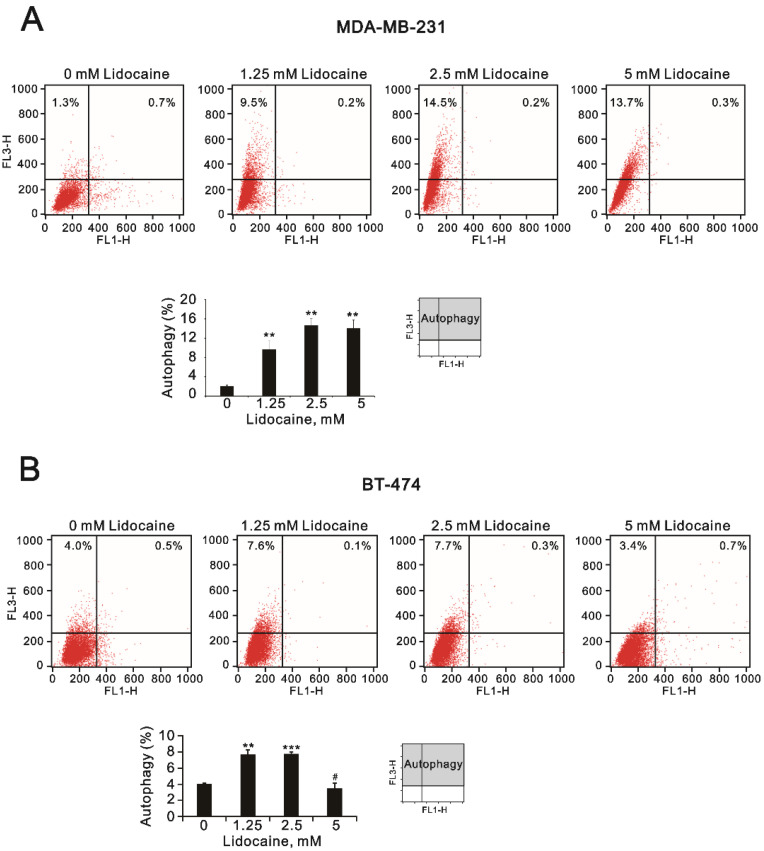
The effects of lidocaine on autophagy in MDA-MB-231 and BT-474 cells. Acidic vesicular organelles were detected and quantified using acridine orange staining and measured using flow cytometry analysis; (**A**) MDA-MB-231 and (**B**) BT-474 (3 × 10^5^) cells were treated with the indicated concentrations of lidocaine (1.25, 2.5, and 5 mM) for 24 h. Acridine orange (1 µg/mL) staining was used to identify autophagic cells via FACS. The intensity of the red fluorescence (y-axis, FL3-H) was proportional to the degree of acidity and the volume of acidic vesicular organelles, including autophagic vacuoles. The values refer to the percentages of cells with a significant proportion of acidic vesicular organelles. The results are representative of two independent experiments. Bars depict the mean ± SD. # *p* > 0.05; ** *p* < 0.01; *** *p* < 0.001.

**Figure 10:**
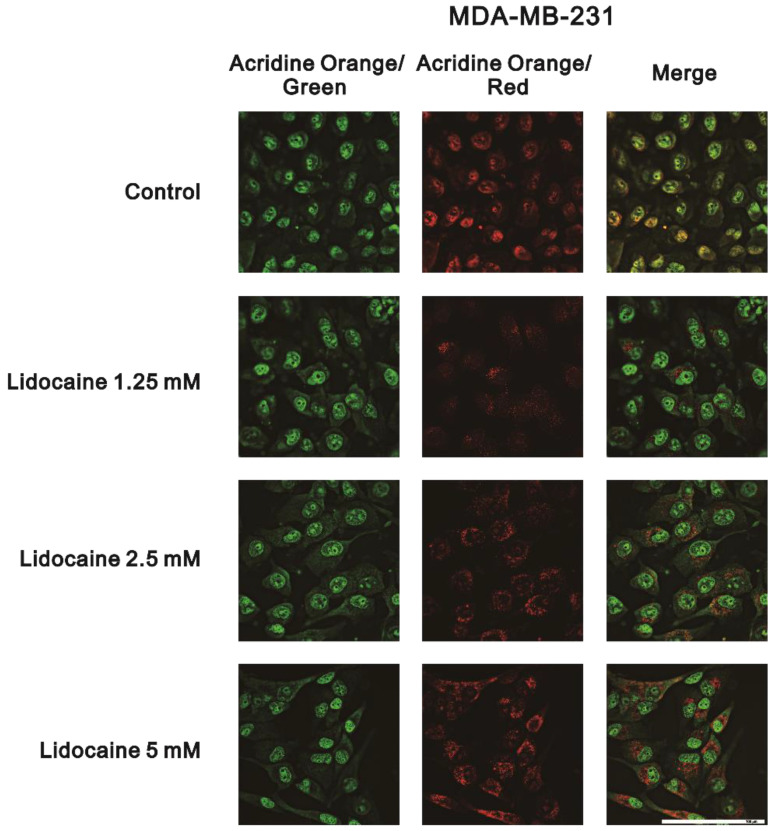
The detection of lidocaine-induced acidic vesicular organelles using acridine orange staining. MDA-MB-231 (3 × 10^5^) cells were treated with the indicated concentration of lidocaine for 48 h before being stained with 1 µg/mL of acridine orange. The formation of acidic vesicular organelles was examined via fluorescence microscopy. Acridine orange is a weak base that accumulates in acidic spaces and results in bright red fluorescence (punctuation) in the cytoplasm, which can be detected by fluorescence microscopy. Scale bar = 100 µm.

In addition to its stronger synergy with doxorubicin (decreased from 0.75 µM to 0.06 µM) in MDA-MB-231 cells, lidocaine is the most commonly used local anesthetic. Hence, we further examined the mechanistic pathways of lidocaine that are involved in the formation of autophagy in MDA-MB-231 and BT-474 cells. Acridine orange has been described as being trapped in high concentrations of acidic vesicles [36]. A green fluorescence emission is observed from the monomeric form of acridine orange and a red fluorescence emission is attributed to the stacking of acidic vesicular organelles. Here, we quantified acidic vesicular organelles using flow cytometry to measure the effects of lidocaine on the formation of autophagolysosomes. In MDA-MB-231 cells, lidocaine significantly induced the formation of autophagolysosomes in a dose-dependent manner (Figure 9A). The effects of lidocaine-induced autophagolysosome formation increased and then decreased compared to the control level in BT-474 cells (Figure 9B). Confocal images of acidic vesicular organelles were observed in MDA-MB231 cells, which were induced by lidocaine (Figure 10). The areas and amounts of oligomeric structures in acridine orange acid vesicles increased with the increasing concentrations of lidocaine in MDA-MB-231 cells (Figure 10; red dots compared to green dots).

**Figure 11:**
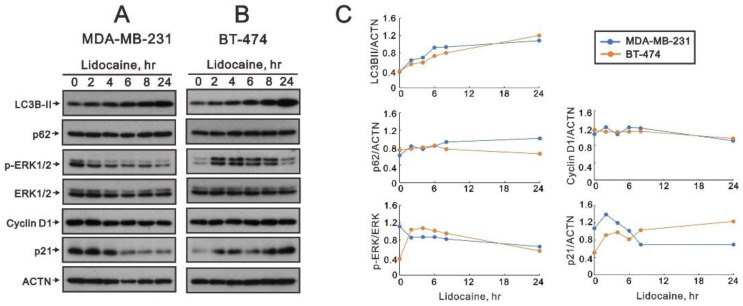
The effects of lidocaine on cell signaling regulation in MDA-MB-231 and BT-474 cells. (**A**) MDA-MB-231 and (**B**) BT-474 (3 × 10^5^) cells were treated with 2.5 mM of lidocaine for the indicated time points. The cell lysates were subjected to Western blotting analysis using antibodies against the indicated proteins. ACTN was used as the protein loading control. (**C**) The protein bands (**A**,**B**) were quantified through pixel density scanning and evaluated using ImageJ, version 1.44a (http://imagej.nih.gov/ij/) (accessed on 1 November 2022). The ratios of protein/ACTN and pERK/ERK were plotted.

We further examined proteins associated with the induction time of autophagy formation by lidocaine in MDA-MB-231 and BT-474 cells. Our Western blotting data showed increasing trends in LC3BII and p62 and decreasing trends in the p-ERK/ERK ratio, cyclin D1, and p21 in MDA-MB-231 cells (Figure 11A,C). Increasing trends were observed in LC3BII, the p-ERK/ERK ratio, and p21 and decreasing trends in p62 and cyclin D in BT-474 cells (Figure 11B,C).

## 3. Discussion

Breast cancer is one of the most prevalent malignancies in women, exceeding lung cancer for the first time in 2020. Operations are usually the first form of breast cancer treatment. Lidocaine is the most commonly used local anesthetic and it effectively inhibits the invasion of cancer cells in concentrations that are used in surgical procedures and cell line studies [11,12,16]. The limited treatment options for TNBC represent unmet clinical needs. A high concentration (>1 mM) of local anesthetic applied to either MDA-MB-231 or MCF7 cells for 48 h significantly inhibited cell viability and induced cytotoxicity; however, at plasma concentrations (~10 μM) for 72 h, no local anesthetic affected cell viability or migration in either cell line [37]. In this study, we observed that four local anesthetics, lidocaine, levobupivacaine, bupivacaine, and ropivacaine, were cytotoxic to two breast tumor cell lines, MDA-MB-231 and BT-474. In addition to the induction of apoptosis and the suppression of the cellular proliferation rate, the four local anesthetics decreased the levels of ROS and increased the elongation of the autophagy biomarker (i.e., the ratio of LC3B II/I) in both cells. Our combination index analysis with doxorubicin showed that ropivacaine had a synergistic effect in MDA-MB-231 and BT-474 cells, and lidocaine had a synergistic effect only in MDAMB-231 cells. There were had no synergistic effects of doxorubicin. We further dissected the different effects of lidocaine on autophagy initiation and autophagosome formation in MDA-MB-231 and BT-474 cells using the autophagy inhibitor 3-MA and acridine orange, respectively. Regardless of the presence of lidocaine, 3-MA decreased the ratio of LC3B II/I in MDA-MB-231 cells and decreased the amount of LC3B II in BT-474 cells. Lidocaine contributed significantly to the formation of autophagolysosomes via the measurement of acridine orange acidic vesicle amounts in a dose-dependent manner in MDA-MB-231 cells but not in BT-474 cells. Our Western blotting analysis of the lidocaine induction time showed decreasing trends in the p-ERK/ERK ratio, cyclin D1, and p21 in MDA-MB-231 cells, but the effects of lidocaine were different in BT-474 cells. This different responsiveness to MDA-MB-231 and BT-474 cells was supported by the synergistic effect of doxorubicin and the association of autophagy elongation and autophagolysosome formation in MDA-MB-231 cells.

Local anesthetics primarily work on sodium channels to block the sodium ion permeation over the cell membrane and on different channels for calcium and potassium [26]. All local anesthetic agents can produce central nervous system (CNS) toxicity, decreased myocardial contractility, and potentially fatal cardiac arrhythmias from clinical doses [38,39]. The incidence of local anesthetic systemic toxicity is 1–11 cases per 10,000. Infants and patients with decreased liver function and low cardiac output are vulnerable to systemic toxicity. For prevention of local anesthetic systemic toxicity, small-dose, divided administration, using agents with low toxicity, such as ropivacaine and levobupivacaine, and performing an aspiration test are recommended. The localized delivery of local anesthetics directly to the tumor tissue might reduce the risk of systemic toxicity in the heart or the brain. A similar effect of local anesthetic agents on autophagic responses in primary neurons and the regulation of autophagy by local anesthetic agents is cell-type dependent [40]. The local anesthetic lidocaine can, directly and indirectly, affect different cancers. The direct mechanisms are inhibiting proliferation and inducing apoptosis and the indirect influences are immune regulation, anti-inflammation, and postoperative pain relief [41]. Partner relationships between anticancer drugs and local anesthetics urgently need to be developed to increase the efficacy of anticancer drugs and decrease the side effects of anticancer drugs and local anesthetics for cancers [42]. The challenge is to decrease the concentration of local anesthetics used for anticancer purposes.

Earlier research reported that bupivacaine inhibits angiogenesis by inhibiting the AKT/mTOR pathway and activating the MAPK pathway [43]. The mTOR pathway makes a negative contribution to autophagy by regulating autophagy proteins and lysosome biosynthesis. The primary function of autophagy is to promote cell survival following stress or nutrient limitation by recycling essential cellular components [33,34]. However, in specific contexts, autophagy also leads to cell death; however, the physiological roles and molecular mechanisms of autophagy-dependent cell death have been less well characterized [11,13,16]. In addition to apoptosis and necrosis, the roles of autophagy in cell death can be defined as (i) autophagy-associated cell death; (ii) autophagy-mediated cell death; and (iii) autophagy-dependent cell death [16,36]. However, 3-MA is a nonspecific inhibitor at millimolar concentrations and may affect other targets, including class I PI3K (which inhibits autophagy) and the mitochondrial permeability transition pore (which is a decisive event in both apoptosis and necrosis). It would be interesting to determine whether the functional roles of lidocaine or other local anesthetics involve apoptosis via the modulation of autophagy to produce cytotoxic effects on human breast cancer cells.

BT-474 cells highly express HER2 proteins, which can activate a cascade in the RAS/RAF/MEK/ERK pathway that promotes tumorigenesis [44]. Our data showed that lidocaine produced time-dependent ERK activation in BT-474 cells and time-independent ERK repression in MDA-MB-231 cells. The difference in ERK activation by lidocaine between MDA-MB-231 and BT-474 cells could be the primary cue to explain our differential findings, including the synergy with doxorubicin, the effects on LC3B II and the LC3B II/I ratio, and the regulation of p21 and cyclin D1 expression. ROS have been shown to activate corresponding pathways for the activation of the ERK pathway [45]. One study showed that ROS formation was inhibited by the ERK1/2 pathway inhibitor U0126 [46]. Here, the local anesthetics decreased the cytosolic ROS levels in MDA-MB-231 and BT-474 cells, suggesting that they could disrupt the homeostasis of ROS. The crosstalk between the homeostasis of ROS and the ERK signaling pathway needs further investigation to understand the cytotoxicity and autophagy caused by local anesthetics in human breast cancers.

Some retrospective studies have shown that the use of local anesthesia can improve clinical outcomes after cancer surgeries, including for breast cancers [5,47,48]. However, a recent clinical prospective trial showed that paravertebral blocks did not reduce breast cancer recurrence after potentially curative surgeries [49]. On the other hand, regional anesthesia modalities can produce clinically significant decreases in pain scores and cumulative opioid consumption compared to other anesthesia techniques [8,50,51]. Therefore, regional anesthesia using local anesthetics during cancer resection surgeries may enhance short-term recovery and may influence long-term outcomes, which has spawned the global emergence of the subspecialty of onco-anesthesia [52]. Meanwhile, two studies demonstrated that lidocaine inhibited cancer migration and enhanced the efficacy of the cyclin-dependent kinase 4/6 inhibitor palbociclib in TNBC [12,53]. Moreover, local anesthetics, including lidocaine, can be administered intravenously to manage refractory cancer pain and have a direct action on cancer cells [18,37,54]. Lidocaine and ropivacaine (but not bupivacaine or demethylated deoxyribonucleic acid) combined with the anticancer drug 5′-aza-2′-deoxycytidine can exert synergistic demethylating effects on the suppression of tumor growth [55]. Our data showed the synergistic effect of lidocaine with doxorubicin on MDA-MB-231 cells and that of ropivacaine with doxorubicin on MDA-MB-231 and BT-474 cells, suggesting that the local anesthetic agent should be carefully selected according to the breast cancer cells for effective combinatory therapy. Further investigations are needed to prove the benefits of continuous lidocaine infusion, regional anesthesia using lidocaine during breast cancer surgeries, lidocaine infusion during chemotherapy with doxorubicin, and treating TNBC patients with cancer pain with lidocaine patches.

## 4. Materials and Methods

### 4.1. Cell Cultures and Reagents

The human MDA-MB-231 TNBC cells, human breast cancer BT-474 cells, and human normal gingival epithelial SG cells were cultured in Roswell Park Memorial Institute (RPMI) 1640 medium (Corning, Corning, NY, USA), containing 10% fetal bovine serum (FBS) and 1% penicillin–streptomycin (Invitrogen, Waltham, MA, USA). The lidocaine, bupivacaine, levobupivacaine, ropivacaine, acridine orange, and doxorubicin were purchased from Sigma–Aldrich (St. Louis, MO, USA). The autophagy inhibitor 3-methyadenine (3-MA) was purchased from InvivoGen (San Diego, CA, USA).

### 4.2. Metabolic Activity Analysis

Cells were plated into 24-well culture plates and incubated for 1 day, after which fresh RPMI 1640 medium containing the selected drugs was added to each well. The procedural details have been described in our previous publications [56,57]. The cells were incubated with the selected treatments for the indicated periods. Then, the cells were incubated in MTT (thiazolyl blue tetrazolium bromide) solution (0.5 mg/mL) for 1 h at 37 °C. At the end of the reaction, DMSO was added to dissolve the formazan crystals that formed from the reaction. The absorbances at 570 nm and 650 nm were measured using a multimode microplate reader (Varioskan™ LUX, Thermo Scientific™, Waltham, MA, USA). The metabolic activity was calculated based on the absorbance ratio between the cells cultured with the selected drugs and the untreated controls, which were assigned a value of 100. The combination index (CI) was calculated utilizing CalcuSyn (Biosoft, Cambridge, UK) to generate isobolograms. Typically, a CI value <1 denotes a synergistic combination effect and a CI value >1 denotes an antagonistic combination effect.

### 4.3. Western Blotting Analysis

Cells were lysed with lysis buffer (100 mM tris–HCl (pH 8.0), 150 mM NaCl, 0.1% SDS, and 1% Triton 100) at 4 °C. The protein concentrations in the lysates were measured using Bio-Rad Protein Assay Dye Reagent Concentrate (Bio-Rad Laboratories, Hercules, CA, USA). The protein lysates were prepared with 4× protein loading dye and denatured at 95 °C for 10 min, separated on 12% SDS-PAGE, and blotted onto PVDF membranes (Immobilon-P; Millipore, Bedford, MA, USA) using a Bio-Rad Semi-Dry Transfer Cell. The blots were then incubated with the primary antibodies against α-actinin (ACTN, H-2, sc-17829, 1:5000) and p62 (D-3, sc-28359, 1:1000 dilution) (Santa Cruz Biotechnology, Santa Cruz, CA, USA); LC3B (#2775, 1:1000 dilution), p-ERK1/2 (#4370, 1:1000 dilution), ERK1/2 (#4695, 1:1000 dilution), cleaved PARP (Asp214) (#9546, 1:1000 dilution), cleaved Caspase-3 (Asp175) (#9664, 1:1000 dilution), ATG5 (#12994, 1:1000 dilution), and BCL-2 (#2876, 1:1000 dilution) (Cell Signaling, Danvers, MA, USA); cyclin D1 (ab134175, 1:1000 dilution) and p21 (ab109520, 1:1000 dilution) (Abcam, Cambridge, UK). Thereafter, the blots were incubated with HRP-conjugated secondary antibodies (anti-mouse IgG (AP192P) and anti-rabbit IgG (AP132P), Merck-Millipore). The immunoreactive proteins were detected using ECL™ Western Blotting Detection Reagent and Amersham Hyperfilm™ ECL (GE Healthcare, Waukesha, WI, USA). The procedural details have been described in our previous publications [56,57].

### 4.4. Fluorescence-Activated Cell Sorting (FACS) for Flow Cytometry Analyses of Cell Cycle Profiles, Proliferation, and ROS

The cell cycle profiles were measured according to their cellular DNA content using FACS. Briefly, the cells were seeded in 6-well culture plates and treated with the selected drugs for 24 h before being harvested. The cells were fixed, permeabilized, and stained with 7-Aminoactinomycin (7-AAD, BD Biosciences, San Jose, CA, USA). The cell cycle distribution was then evaluated using FACS, based on the cellular DNA content. The cell proliferation was assessed using immunofluorescent staining with incorporated bromodeoxyuridine (BrdU) (BD Pharmingen™ BrdU Flow Kit) and flow cytometry, according to the manufacturer’s instructions. Briefly, the cells were seeded in 6-well culture plates and treated with the selected drugs for 24 h. After incubation, the cells were stained with BrdU, harvested, washed with PBS, and then fixed and permeabilized before being stained BrdU with fluorescent antibodies. The cells were resuspended in staining buffer and an FITC-BrdU fluorescence analysis was performed using a FACSCalibur flow cytometer and Cell Quest Pro software (BD Biosciences). The intracellular ROS levels were determined using the fluorescent marker DCFH-DA. Briefly, the cells were treated with the selected drugs for 24 h, stained with DCFH-DA (20 μM) for 40 min at 37 °C, and then harvested. Afterwards, the cells were washed once with PBS, and then the DCFH-DA fluorescence intensity was analyzed on the FL-1 channel of the FACSCalibur flow cytometer using Cell Quest Pro software (BD Biosciences). The median fluorescence intensity of the vehicle was used as the starting point for M1 gating.

### 4.5. Flow Cytometric Quantification of Acidic Vesicular Organelles

The acidic compartments of the cells were detected using acridine orange (Sigma, Cat. No. A8097) staining and were measured by flow cytometry. As the protonated form of acridine orange accumulates inside acidic vesicles, it is a marker for the final steps of the autophagy process. Briefly, the cells were treated with the indicated lidocaine dosages for 24 h, stained with acridine orange (1 μg/mL) for 20 min at 37 °C, and then trypsinized for harvesting. Afterwards, the cells were washed once with PBS, resuspended in 400 μL of PBS, and then analyzed via flow cytometry (FACSCalibur, BD, Biosciences). The excitation wavelength was 488 nm, and fluorescence was detected at 510–530 nm (green fluorescence, FL1) and 650 nm (red fluorescence, FL3). The data were analyzed using the CellQuest™ software program. The percentage of autophagy cells was calculated based on the number of cells present in the upper-left and upper-right quadrants.

### 4.6. Detection of Acidic Vesicular Organelles Using a Fluorescence Microscope

The formation of cell acidic compartments, which is a morphological characteristic of autophagy, was detected using acridine orange staining. Briefly, the cells were cultured in 12 mm microscope cover glasses and treated with the indicated lidocaine dosages for 24 h and then stained with 1 μg/mL of acridine orange for 15 min. The samples were observed under a Leica DMi8 S Thunder widefield fluorescence microscope (Leica Microsystems, Wetzlar, Germany) using the emission wavelengths for green and red fluorescence, i.e., 506~532 nm and 578~610 nm, respectively. Acridine orange is a weak base that accumulates in acidic spaces and results in bright red fluorescence in the cytoplasm, which can be detected via fluorescent microscopy.

### 4.7. Statistical Analyses

The values are expressed as the mean ± SD of at least three independent experiments. All comparisons between groups were conducted using unpaired two-tailed t-tests. The statistical significance was set at *p* < 0.05.

## 5. Conclusions

Our study demonstrated that four local anesthetics, lidocaine, ropivacaine, levobupivacaine, and bupivacaine, could not only reduce tumor growth and proliferation but also promote apoptosis and autophagy, demonstrating their potential as extremely useful adjunct therapies for breast cancer treatment. Further studies are required to provide more solid evidence that can establish relationships between individual local anesthetic agents, such lidocaine and ropivacaine, and clinical outcomes for various types of breast cancer surgery in order to accentuate the importance of local anesthetics in human breast cancers due to their cytotoxicity mediated through the induction of autophagy.

## Data Availability

Not applicable.
